# A Multi-Mode Broadband Vibration Energy Harvester Composed of Symmetrically Distributed U-Shaped Cantilever Beams

**DOI:** 10.3390/mi12020203

**Published:** 2021-02-16

**Authors:** Xiaohua Huang, Cheng Zhang, Keren Dai

**Affiliations:** School of Mechanical Engineering, Nanjing University of Science and Technology, Nanjing 210094, China; huangxiaohua@njust.edu.cn (X.H.); zhangcheng4432@njust.edu.cn (C.Z.)

**Keywords:** energy harvesting, vibration, broadband, resonant frequency

## Abstract

Using the piezoelectric effect to harvest energy from surrounding vibrations is a promising alternative solution for powering small electronic devices such as wireless sensors and portable devices. A conventional piezoelectric energy harvester (PEH) can only efficiently collect energy within a small range around the resonance frequency. To realize broadband vibration energy harvesting, the idea of multiple-degrees-of-freedom (DOF) PEH to realize multiple resonant frequencies within a certain range has been recently proposed and some preliminary research has validated its feasibility. Therefore, this paper proposed a multi-DOF wideband PEH based on the frequency interval shortening mechanism to realize five resonance frequencies close enough to each other. The PEH consists of five tip masses, two U-shaped cantilever beams and a straight beam, and tuning of the resonance frequencies is realized by specific parameter design. The electrical characteristics of the PEH are analyzed by simulation and experiment, validating that the PEH can effectively expand the operating bandwidth and collect vibration energy in the low frequency. Experimental results show that the PEH has five low-frequency resonant frequencies, which are 13, 15, 18, 21 and 24 Hz; under the action of 0.5 g acceleration, the maximum output power is 52.2, 49.4, 61.3, 39.2 and 32.1 μW, respectively. In view of the difference between the simulation and the experimental results, this paper conducted an error analysis and revealed that the material parameters and parasitic capacitance are important factors that affect the simulation results. Based on the analysis, the simulation is improved for better agreement with experiments.

## 1. Introduction

With the development of information technologies and big data technology, wireless sensor nodes and portable electrical devices have been widely used in many fields, such as industry, healthcare and agriculture [1,2]. At present, the service life of these devices is severely limited due to the limited power and capacity loss of the battery. At the same time, the cost of replacing the battery also further restricts the use of these devices. Therefore, the question of how to harvest energy from the surrounding environment to power these devices, instead of simply using batteries, has attracted widespread attention [3,4,5,6]. There are many forms of energy in the natural environment, such as solar energy, wind energy, vibration energy and thermal energy [7,8,9,10], and the commonly used energy harvesting mechanisms include photoelectric, piezoelectric [11,12], electromagnetic [13,14,15], electrostatic [16] and triboelectric effects [17,18]. In particular, there are abundant vibration energy sources in the natural environment, most of which are low-frequency vibration [19]. Piezoelectric energy harvesters (PEH) are widely used for vibration energy collection due to their large power density, simple structure and ease of fabrication [20,21].

Traditional PEHs commonly have a cantilever structure, which only generates power within a limited vibration frequency range near the resonance frequency of the first mode. When the vibration frequency of the vibration source in the surrounding environment deviates from the first resonance frequency, the output power of the energy harvester will drop sharply, resulting in a low energy harvesting efficiency and limited application scenarios.

In order to solve this problem, various methods have been proposed to broaden the operation bandwidth of the PEH. One of the solutions is to build a piezoelectric cantilever array with different sizes and resonant frequencies [22,23], which can also harvest more energy. However, the cantilever array results in a relatively larger volume, which weakens the output power density of the device. Another solution is to design an energy harvester with a single tip mass, which can generate a single resonance frequency over which maximum energy can be harvested. Therefore, the resonance frequency can be adjusted by adjusting the properties of the tip mass, such as the center of gravity of the proof mass. Based on this advantage, auto-tuning or self-tuning devices consisting of hollow masses that encapsulate moving cylinders inside were realized [24,25,26]. With the change in input excitation, the moving cylinder will occupy a new position inside the hollow mass, resulting in a new resonance frequency. Unfortunately, the structures of these devices are too complicated due to the additional tuning component. Moreover, nonlinear techniques, such as bi-stable systems [27,28] and Duffing oscillation [29,30,31], can effectively improve the frequency response with a broad bandwidth, but nonlinear vibration of the harvester would happen only when the environmental vibration intensity is strong enough to induce the stretching force in the beam of the energy harvester.

Recently, researchers have proposed the idea of multiple-degrees-of-freedom (DOF) PEH to realize multiple resonant frequencies within a certain range to achieve broadband vibration energy harvesting. Wu et al. [32] proposed a 2-DOF PEH composed of a main beam and a second beam, which is cut out inside the main beam. The two resonant frequencies can be tuned to be closer to each other by changing the tip masses. Sun and Peter [33] designed an asymmetrical U-shaped PEH to obtain two resonant frequencies which are close to each other. Further, Zhang and Hu [34] made use of the branching cantilever structure and proposed a harvester consisting of a main beam with a piezoelectric layer and several branched beams with tip masses at their free ends. The number of resonance frequencies can be increased by increasing the number of branch beams. 

In this paper, a multi-mode broadband PEH is proposed. The harvester is composed of five tip masses, two U-shaped cantilever beams and a straight beam. The bandwidth of the harvester can be widened by adjusting the weight of the tip masses and the length of the cantilever beam. Simulation and experimental study are carried out to prove its validation for wideband energy harvesting. An error analysis is also carried out to reveal the impact of material parameters and parasitic capacitance on output performance, and we improved the simulation for better agreement with the experiment.

## 2. Structural Design and Fabrication

### 2.1. Structural Design 

**Figure 1:**
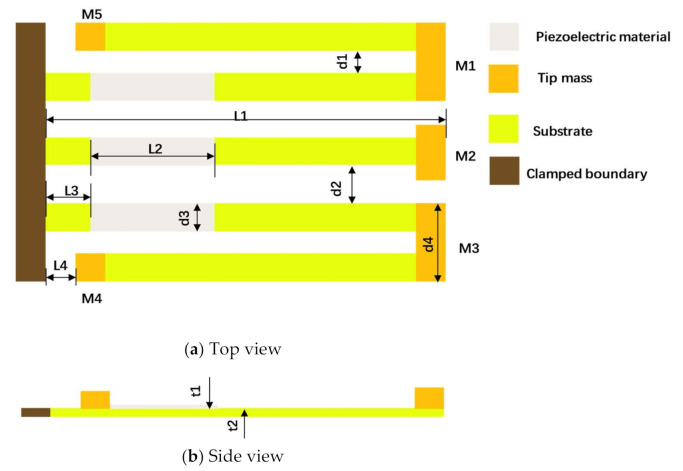
Schematic of the proposed PEH. (**a**) Top view; (**b**) Side view.

The schematic of the proposed multi-mode PEH is shown in Figure 1. As has been studied in [34], the U-shaped cantilever with two tip masses can narrow the frequency band gap between the first two resonant frequencies. Therefore, the PEH in this paper is designed to be composed of two symmetrically distributed U-shaped cantilever beams, a straight beam with three piezoelectric layers bonded on them and five tip masses. Among the five tip masses, one is attached to the free end of the straight beam, and the other four are attached to the turn-back and free end of the two U-shaped cantilever beams. The 5 resonance frequencies close to each other within a certain range are realized by specific arrangement of the multiple tip masses, and the geometric parameters are shown in Table 1.

### 2.2. Fabrication 

**Figure 2:**
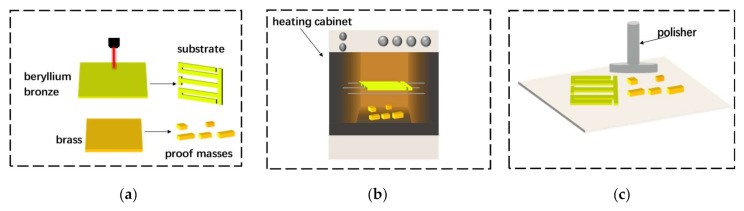

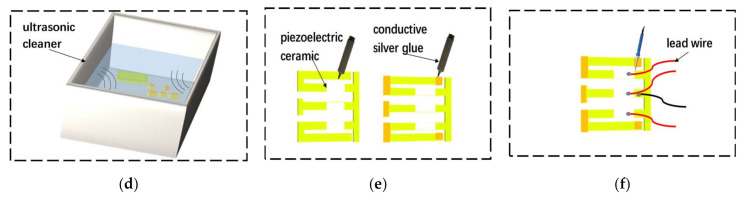
The fabrication processes of the proposed PEH. (**a**) Laser cutting; (**b**) Surface heating; (**c**) Surface polishing; (**d**) Surface cleaning; (**e**) Bonding piezoelectric ceramics and proof masses; (**f**) Wire bonding.

The entire fabrication process of the PEH is shown in Figure 2. The detailed processing procedure is as follows:

(a) Laser cutting. A laser cutting machine is used to cut the substrate and five tip masses according to the designed geometric dimensions.
(b) Surface heating. In order to make the surface of the metal substrate smooth, it needs to be placed in a heating box, heated at 300℃ for two hours and then taken out and cooled to room temperature.
(c) Surface polishing. Place the cooled metal substrate and tip masses on a polishing machine and use the polishing machine to remove impurities and oxide layers on the surface of the substrate and five tip masses to ensure a clean and flat surface.
(d) Surface cleaning. Put the polished metal substrate and tip masses into an ultrasonic cleaning machine to clean the surface of impurities to facilitate subsequent bonding work. Put the cleaned parts on a clean glass plate and let them dry.
(e) Bonding piezoelectric ceramics and proof masses. The piezoelectric ceramic sheet and the metal substrate need to be connected with conductive silver glue to meet the requirements of mechanical connection and electrical connection. Place the piezoelectric ceramic sheet and the metal substrate on a flat operating table, use a small brush to evenly spread the conductive silver glue on the surfaces of both and then place the piezoelectric ceramic on the metal substrate and press it gently. Wipe gently with a cotton ball dipped in acetone solution to remove excess glue. Similarly, the masses are bonded. After the piezoelectric ceramics and the mass block are bonded, place the bonded structure for more than 24 h to ensure a stable bonding.
(f) Wire bonding. Place the energy harvester on the operating table, and use an electric soldering iron to weld the thin wires on the three electrodes and the upper surface of the metal substrate, respectively, as the positive and negative electrodes of the energy harvester. Then, use conductive silver glue to bond the three electrodes on the surface of the piezoelectric ceramic; the three wires on the three electrodes are connected in parallel.

## 3. Simulation and Experimental Study

In order to verify the performance of the designed PEH, a finite element simulation and experiment are carried out.

### 3.1. Simulation Study 

All simulation studies on the proposed PEH are carried out in the finite element simulation software, the COMSOL Multiphysics (COMSOL, Stockholm, Swede). This is a multi-physical field coupling analysis software and provides a dedicated module that can be used to simulate piezoelectric transducers. The dimensions of the finite element model are set according to Table 1. The three piezoelectric ceramics attached to the substrate are connected in parallel; the surface of three piezoelectric ceramics and the substrate are connected to the two terminals of the load resistor, respectively. Thus, the finite element model of the proposed PEH connected with the load resistor is established. Modal analysis is carried out to determine the first six vibration modes and resonance frequencies of the PEH. Harmonic excitation is performed in modal analysis to obtain the voltage response and the optimal matching resistance of the PEH. The simulation parameters of the proposed PEH are shown in Table 2.

**Figure 3:**
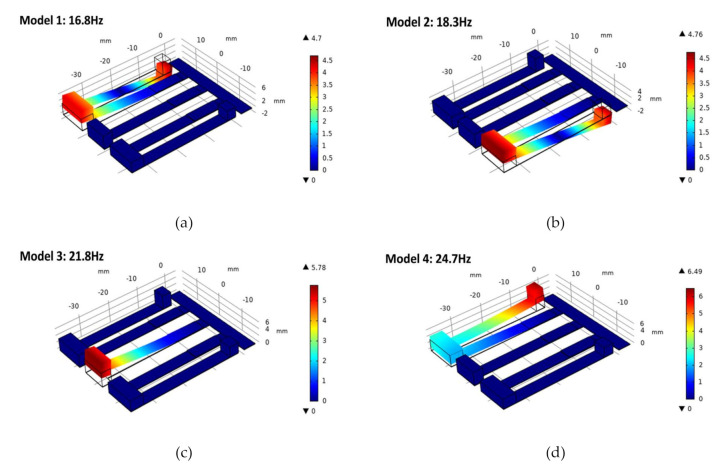

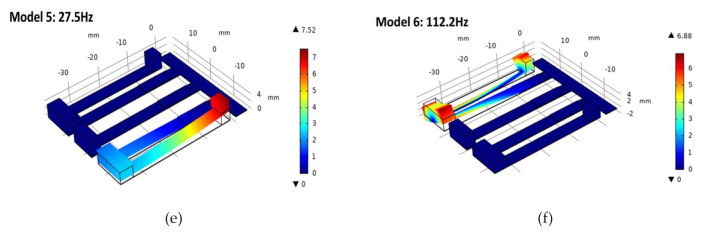
(**a**–**f**) Finite element simulation of multiple mode shapes of the proposed structure.

The first six vibration modes are shown in Figure 3; the resonance frequencies are 16.8, 18.3, 21.8, 24.7, 27.5 and 112.2 Hz. In the first five vibration modes, the deformation of the PEH is the motion of the cantilever beam along the direction of substrate thickness. In the sixth vibration mode, the cantilever beam is twisted; at the same time, its resonance frequency is much higher than the first five resonance frequencies. Therefore, only the first five resonant modes of the PEH are studied in this paper. In Figure 3, the motion of tip masses M1, M2 and M3 forms the first three resonant frequencies, respectively. The fourth and fifth resonant frequencies are caused by tip masses M4 and M5. The movement of the masses M4 and M5 drives the vibration of the masses M1 and M3, which causes the up and down vibration of the cantilever beam. Therefore, each tip mass determines the resonance frequency of the PEH. By adjusting the weight of tip masses, the resonance frequencies of the PEH can be changed, so the bandwidth of the PEH can be widened by shortening the interval between different resonance frequencies.

**Figure 4:**
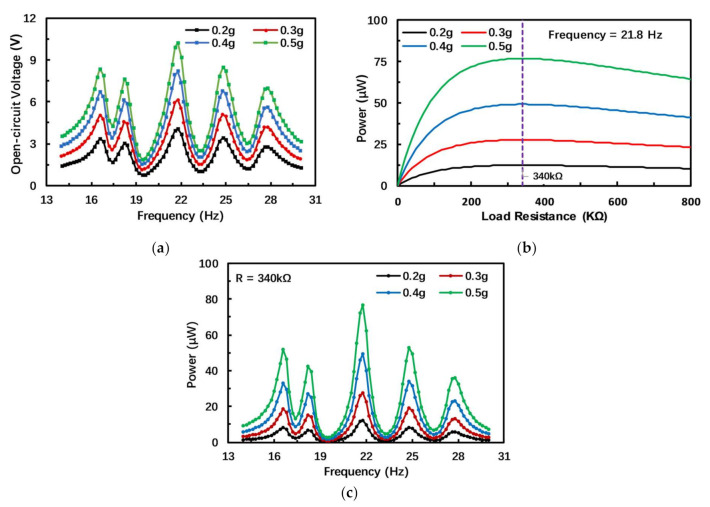
Simulation results of the output characteristics of the PEH. (**a**) Simulation results of frequency response of open-circuit voltage; (**b**) Simulation results of output power against load resistance under different acceleration amplitudes; (**c**) Simulation results of frequency response of output power.

Modal analysis has shown that the PEH has five resonance frequencies in the low-frequency range. Frequency response analysis is performed to confirm whether the output voltage of the PEH is improved by coupling resonance frequencies. By adjusting the resistance to an extremely large value, the PEH can be considered in open-circuit condition. The frequency response of the open-circuit voltage is shown in Figure 4a. When the acceleration is 0.5 g, the proposed harvester has five voltage peaks; maximum peak voltages are 8.5, 7.6, 10.2, 8.4 and 7.1 V. It is validated that the structure of the PEH can generate multiple resonant voltages by modal coupling. According to the simulation result, as the acceleration increases, the output voltage of the PEH also increases; when the frequency deviates from the resonance frequencies, the output voltage drops rapidly. Therefore, if the frequency intervals between voltage peaks continue to expand, the operating bandwidth will decrease sharply. In this work, the frequency interval shortening mechanism is used to increase operation bandwidth.

The output power is an important indicator for evaluating the performance of a PEH. The PEH is equivalent to a power supply to provide energy to the load, and the load can be simply viewed as a resistor. In order to obtain the maximum output power from the PEH and improve the working efficiency of the PEH, the optimal load resistance value of the PEH need to be determined based on the principle of impedance matching. Therefore, the relationship between the output power and the load resistance is studied. Although the optimal resistance value at each frequency will be different, the output power near the resonance frequency is the largest and the most noteworthy. Therefore, the optimal resistance at the resonance frequency is the most valuable to study. Since the output voltage of the PEH is the largest at the third resonance frequency, this article seeks the optimal resistance value at the third-order resonance frequency. 

The relationship between output power and load resistance is shown in Figure 4b (the acceleration amplitude changes from 0.2 to 0.5 g with an interval of 0.1 g). In the simulation study, the variable resistor value increases from 0 to 800 kΩ in intervals of 10 kΩ. According to the simulation result in Figure 4, as the resistance increases, the output power of the harvester first increases and then decreases. Therefore, the optimal load resistance for the harvester is 340 kΩ, and only in this case can the PEH output maximum power and improve energy harvesting efficiency.

In order to analyze the output power of the PEH at different frequencies, frequency response analysis of the output power is carried out, and simulation results are shown in Figure 4c. The output power of the PEH with the optimal resistor is similar to the open-circuit voltage, with five peaks at similar frequency points in the low-frequency range. At the third resonance frequency, the output power can reach up to 76.88 μW, when the acceleration is 0.5 g. At the resonance frequency, the output power is the maximum; when the working frequency deviates from the resonance frequency, the output power of the PEH will decrease. Due to the small interval between the resonance frequencies, the average output power of the PEH within a certain range of the resonance frequencies could maintain a relatively high level to satisfy practical application.

**Figure 5:**
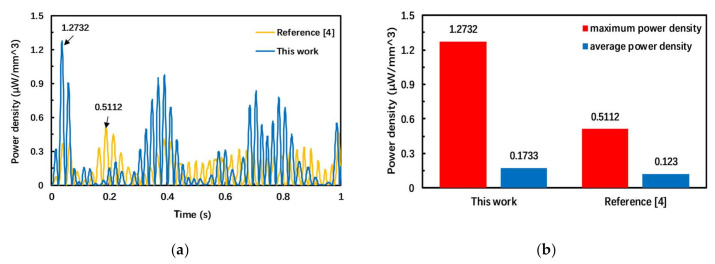
Comparison of output power density of two harvesters. (**a**) Instantaneous output power density during simulation; (**b**) Comparison of maximum and average power density.

Since the geometry sizes of different PEH are different, it is necessary to calculate the power density of the PEH to evaluate its performance. Figure 5 shows the comparison of the power density between this work and an M-shaped folded cantilever PEH [4], when two harvesters are under the action of sinusoidal signals of different frequencies. Consider the operating bandwidth of the two harvesters: the frequency of sinusoidal signals increases from 12 to 28 Hz at an interval of 1 Hz. The acceleration signal actually applied to the PEH is

(1) $$y=\sum_{k=12}^{28}0.05g\sin(2\pi kt)$$

where g is the acceleration of gravity and *t* is the vibration time. The simulation time set in this paper is one second. The volume of piezoelectric material is used to calculate the power density. As shown in Figure 5, the maximum power density of the PEH designed in this paper is 1.2732 μW${\mathrm{mm}}^{3}$, and the maximum power density in [4] is 0.5112 μW${\mathrm{mm}}^{3}$; the maximum power density of this paper is nearly 2.49 times this value. During the whole simulation period, the power density of the proposed PEH is larger than that in [4] for most of the time, and the average power density is 0.1733 and 0.1230 μW${\mathrm{mm}}^{3}$, respectively. The simulation result confirms that the proposed harvester has higher output power both in terms of maximum power density, average power density and, most of the time, instantaneous power density under a complex environment. 

### 3.2. Experimental Study

#### 3.2.1. Experimental Setup

**Figure 6:**
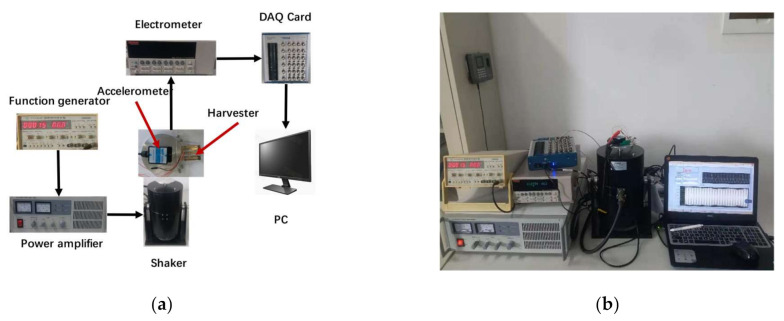
(**a**) Schematic of experimental setup for the PEH testing; (**b**) The experimental scene diagram of the PEH test.

The feasibility of the structure has been verified through simulation, and we next need to build a test platform to test the real object of the PEH. The schematic of the testing system for the proposed PEH is shown in Figure 6. By adjusting the frequency of the harmonic excitation signal of the function generator (YB1602, Lvyang, Yangzhou, China) and the output current of the power amplifier (SA-P050, Shiao, Wuxi, China), the shaker (SA-JZ050, Shiao, Wuxi, China) can generate vibration signals of different frequencies and amplitudes. In the experiment, the excitation frequency is manually swept from 10 to 26 Hz. During this sweeping procedure, the amplitude of the vibration signal generated by the shaker is measured by the accelerometer (HWT901B, Wit, Shenzhen, China) as a feedback loop. Due to the large internal resistance of the PEH, the open-circuit voltage generated by the harvester is measured with an electrometer (Keithley6514, Tektronix, Cleveland, OH, USA), and the measured voltage data are sent to a computer via a data acquisition card (NI USB-6363, National Instruments, Austin, TX, USA).

#### 3.2.2. Experimental Results 

**Figure 7:**
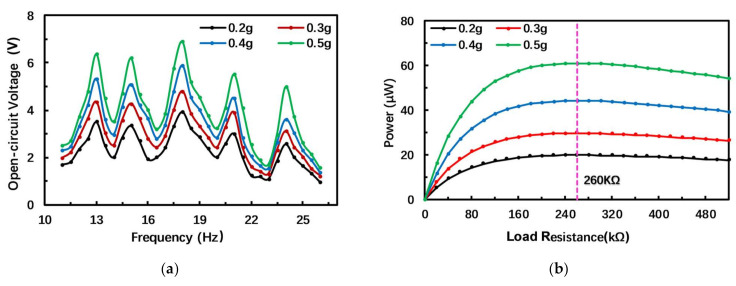

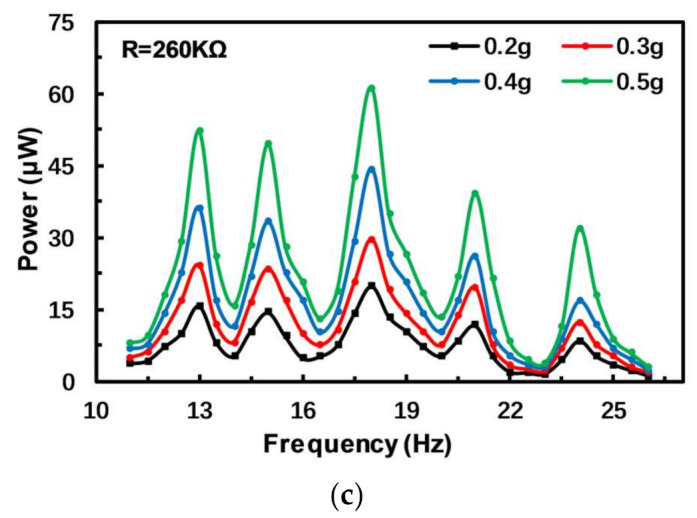
Experimental results of output characteristics of the PEH. (**a**) Measured frequency response of open-circuit voltage; (**b**) Measured results of output power against load resistance under different acceleration amplitudes; (**c**) Measured results of frequency response of output power.

The experiment measured the frequency response of the open-circuit voltage of the PEH under different acceleration amplitudes. The measurement results are shown in Figure 7a. When the acceleration is 0.5 g, the PEH has five voltage peaks in the range of 11–26 Hz, with peak values of 6.4, 6.2, 6.9, 5.1 and 6.4 V, which further confirms the feasibility of the structure of the PEH. The frequency corresponding to the peak voltage is the resonance frequency of the PEH, so the first five resonant frequencies are 13, 15, 18, 21 and 24 Hz. Similarly, the output voltage of the PEH increases with the acceleration amplitude, and the maximum voltage is obtained at the resonance frequency. The output voltage decreases away from the resonance frequency. Compared with the simulation results, the open-circuit voltage and first five resonance frequencies measured by the experiment are smaller than the simulation values.

In order to find the optimal load resistance of the PEH, we studied the relationship between the output power of the PEH and the load resistance. In our experiment, a variable resistor ranging from 10 to 600 kΩ in intervals of 20 kΩ is applied to study the performance with different resistances. The optimal resistance value also appears at the third resonance frequency. Figure 7b shows the relationship between the output power and the load resistance at the third resonance frequency, which is 18 Hz. The output power first increases and then decreases with the increase in resistance. When the load resistance is 260 kΩ, the output power can reach the maximum, approximately 61.3 μW, at an acceleration of 0.5 g, so the optimal resistor value is 260 kΩ for this harvester. Since the output voltage of the PEH is lower than the simulated value, its output power is also lower than the simulated value. Nowadays, many small sensing systems can operate normally with low power consumption. For example, the power consumption of a temperature sensing system in [35] and a pressure measurement microsystem in [36] is only 71 nW and 120 μW (for the whole microsystem), respectively. In addition, these microsystems usually operate at intervals, and the power consumption can be further satisfied with the aid of energy storage and management systems such as super capacitors. Therefore, the proposed PEH can supply these devices directly or through energy storage devices successfully for practical application.

Figure 7c shows the frequency response of the output power of the PEH under different acceleration amplitudes and a load resistance of 260 kΩ. The experimental results and the simulation results display the same changing trend. The output power is the largest at the resonance frequency; when the ambient frequency deviates from the resonance frequency, the output power will decrease.

## 4. Error Analysis

**Figure 8:**
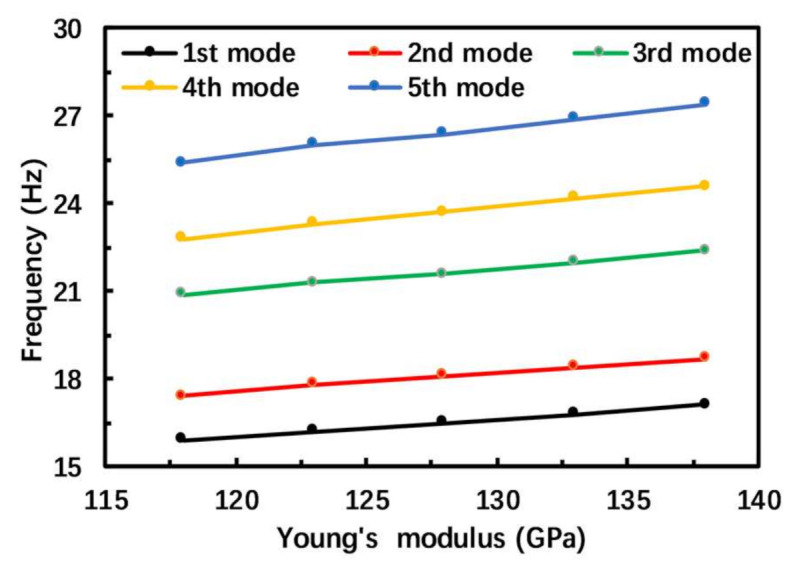
The influence of the Young’s modulus of the substrate on the resonance frequencies.

Simulation and experimental results validate that the PEH does have five resonant frequencies in the low-frequency range, and the variation trend of the open-circuit voltage and output power of the PEH is the same in the simulation and experiment. However, there are some deviations in the values of open-circuit voltage and resonance frequency, and the resonance frequency in the experiment is generally around 3 Hz lower than the simulated value. To further study the source of these deviations, the effects of the Young’s modulus of the substrate on the resonance frequency of the proposed PEH are studied. The influence of the Young’s modulus of the substrate on the resonance frequency is shown in Figure 8. As the Young’s modulus decreases, the first five resonance frequencies also decrease.

**Figure 9:**
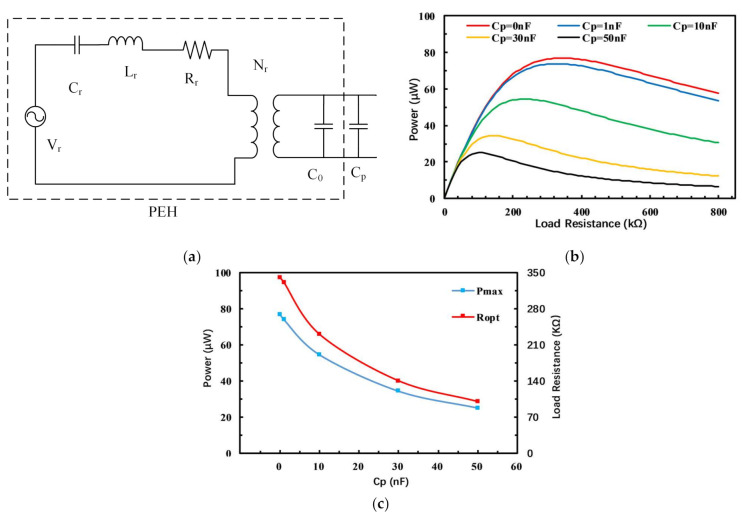
The influence of the parasitic capacitance on output characteristics. (**a**) The equivalent circuit model for the PEH with parasitic capacitances; (**b**) The relationship between output power and resistance for different parasitic capacitances; (**c**) The relationship between maximum output power, optimal matching resistance and parasitic capacitance.

In addition to the Young’s modulus, parasitic capacitance will also have an important impact on the output performance of the PEH [37,38]. The PEH can be regarded as an equivalent circuit network composed of infinite parallel branches composed of inductance, capacitance, resistance, ideal voltage source and ideal transformer, where each parallel branch represents a certain order of vibration mode. The parasitic capacitance usually comes from the internal capacitances of the piezoelectric transducer; at the same time, there are parasitic capacitances in the surrounding environment, such as the external wires, the metal in the environment and capacitances in the measuring equipment. The influence of parasitic capacitance in the environment also needs to be added to the equivalent circuit model. As shown in Figure 9a, the PEH can be represented as an ideal voltage source $V_{r}$ in series with a capacitor $C_{r}$, a resistance $R_{r}$, an inductance $L_{r}$ and finally in parallel with an ideal transformer $N_{r}$. The capacitor $C_{p}$ is the parasitic capacitance and is connected in parallel with the PEH in the equivalent circuit model.

The output characteristics of the PEH for different parasitic capacitances are calculated by simulation, and the results are shown in the Figure 9b. It can be clearly seen that the parasitic capacitance has a strong influence on the output characteristics of the PEH. When the external resistance is small, the output power difference of the PEH is very small, but as the load resistance increases, the larger the parasitic capacitance of the PEH, and the more the output power drops. The relationships between the maximum output power, optimal matching resistance and parasitic capacitance are shown in Figure 9c. As the parasitic capacitance increases, the maximum output power and optimal matching resistance will both decrease rapidly. When the parasitic capacitance increases from 0 to 50 nF, the maximum output power of the PEH is reduced by 62.3% and the optimal matching resistance is changed from 340 to 100 kΩ.

**Figure 10:**
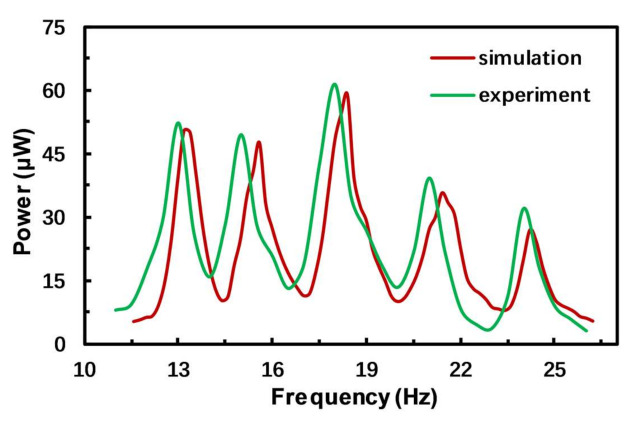
Comparison of simulation and experimental results of frequency response.

Considering the influence of the above factors, the modified parameters (Young’s modulus: 118 GPa, parasitic capacitance: 15 nF) are taken for simulation analysis. It can be seen from Figure 10 that the simulation results using the modified parameters essentially coincide with the experimental results, and the errors are significantly reduced in terms of both output power and resonance frequencies.

## 5. Conclusions

This article proposed a multi-mode broadband PEH composed of symmetrically distributed U-shaped cantilever beams, a straight beam and five tip masses. A piece of piezoelectric ceramic is bonded to each beam. Through structural optimization design, the number of voltage peaks of the PEH device in the low-frequency range is further increased. The experiment and simulation are conducted to validate the feasibility of the proposed structure. The finite element simulation and experiment results show that the PEH can indeed generate five voltage peaks in the range of 10–30 Hz. The power density of the PEH proposed in this article is approximately 1.8 times that of an asymmetric M-shaped cantilever PEH due to its broad working frequency. A theoretical analysis of the error between the idealized modeling, simulation and the non-idealized experimental test results for this type of PEH device reveals the influence of multiple non-ideal factors. The Young’s modulus of the substrate will affect the resonance frequencies of the PEH, and the presence of parasitic capacitance will reduce the maximum output power, frequencies of maximum power and optimal matching resistance. With an adjustment of these parameters, the simulation is improved for better agreement with the experiment.

The proposed PEH realized vibration energy harvesting in a wide frequency range and achieved high power density, indicating its great potential for powering wireless sensor nodes and portable electronic devices in practical application scenarios. The error analysis also provides guidance for the simulation of PEH devices.

## Figures and Tables

**Table 1:** Geometric parameters of the proposed PEH.

Parameter	Value	Parameter	Value
L1	38 mm	t1	0.15 mm
L2	10 mm	t2	0.15 mm
L3	4 mm	M1	10 × 4 × 3${\;\mathrm{mm}}^{3}$
L4	2 mm	M2	8 × 3 × 4${\;\mathrm{mm}}^{3}$
d1	2 mm	M3	10 × 4 × 3.5${\;\mathrm{mm}}^{3}$
d2	5 mm	M4	4 × 3 × 4${\;\mathrm{mm}}^{3}$
d3	4 mm	M5	4 × 3 × 3${\;\mathrm{mm}}^{3}$
d4	10 mm		

**Table 2:** Material parameters of the proposed PEH.

Property	Substrate	Piezoelectric	Tip Mass
Material	Beryllium Bronze	PZT-5H	Brass
Young’s modulus	128 GPa	60.6 GPa	110 GPa
Density	8300 kg/$m^{3}$	7500 kg/$m^{3}$	8500 kg/$m^{3}$
Piezoelectric constant (d31)	-	−2.74 ×$\;10^{-10}\;$C/N	-

## Data Availability

Data are contained within the article.
